# Developing a nursing diagnosis for the risk for malnutrition: a mixed‐method study

**DOI:** 10.1002/nop2.765

**Published:** 2021-01-21

**Authors:** Silvia Brunner, Hanna Mayer, Matthias Breidert, Michael Dietrich, Maria Müller‐Staub

**Affiliations:** ^1^ City Hospital Waid and Triemli, Zurich Zurich Switzerland; ^2^ University Vienna Vienna Austria; ^3^ TU Munich Munich Germany; ^4^ University Zurich Zurich Switzerland; ^5^ Hanze University Groningen Groningen The Netherlands; ^6^ Pflege PBS Wil Switzerland

**Keywords:** 80 and over, aged, interventions and outcomes (Q‐DIO), mixed method, nursing diagnosis, nursing process, protein–energy malnutrition, quality of nursing diagnosis, risk assessment, standardized nursing terminology

## Abstract

**Aim:**

As the risk for malnutrition in older people in hospitals is often underreported, we aimed to develop a risk nursing diagnosis, including label, definition and risk factors.

**Design:**

A convergent parallel mixed‐methods design was employed.

**Methods:**

A literature review led to risk factors, validated by 22 hospitalized older people's perspectives and observations, including their nursing records. Per participant, one interview (qualitative), one non‐participatory observation of three meals (198 hr; qualitative) and one nursing record evaluation (quantitative) were conducted.

**Findings:**

According to the classification system of NANDA International, the risk for protein–energy malnutrition is defined with 18 risk factors, including associated conditions. Content validated risk factors are presented from three participants with the most, medium and least coherent nursing record, measured with the Quality of Diagnosis, Intervention and Outcomes tool.

**Conclusion:**

This new nursing diagnosis supports nurses to manage the risk for malnutrition and optimize older people's nutrition.

## 1 INTRODUCTION

Malnutrition is a major, often unrecognized and underreported problem among older people, especially in hospitals (Leij‐Halfwerk et al., 2019, p. 13). Malnutrition is associated with increased complication rates and healthcare costs (Barker et al., 2011; Volkert et al., 2010). In a multicenter study with hospitalized patients from internal medicine and perioperative wards, the prevalence of malnutrition was 21.4%, while no nutritional screening was conducted in half of the investigated departments (Bonetti et al., 2017). In the present research project, a protein–energy deficiency is defined as at least three points in the nutritional risk screening (NRS‐2002, also called Kondrup Score) (Kondrup et al., 2003) and either an unintentional weight loss of more than 5% in 3 months or an unintentional intake of less than 50%–75% of the required amount in the past week (BFS & Bundesamt für Statistik., 2020).

### 1.1 Background

The risk for malnutrition in older people in acute hospitals was neither systematically nor comprehensively assessed, leading to the omission of necessary interventions (Barker et al., 2011; Haldemann‐Jenni et al., 2016). A primary reason could be that “risk for malnutrition” is missing in the classification of NANDA International, Inc. (NANDA‐I). Therefore, nurses have no tool to recognize the risk for malnutrition and to act accordingly. Nevertheless, this risk is an essential component of inpatients' assessment by nurses. This project focused on older people (aged 80 years and above) in hospitals. Older hospitalized people have an exceptionally high protein requirement and an increased risk for malnutrition and its consequences (DGE, 2015; Nowson & O'Connell, 2015; Singler et al., 2016; Wirth & Volkert, 2017).

#### 1.1.1 Advanced nursing process

The *Advanced Nursing Process* has been explained because it is a scientifically sound overarching concept where nursing diagnoses are embedded. It is an in‐depth nursing process with a comprehensive nursing assessment of the patient's situation, history taking and evidence‐based concepts. Its definition is: “The Advanced Nursing Process consists of defined, validated concepts. It includes assessment, nursing diagnoses, nursing interventions and nursing outcomes that are rooted in scientifically based nursing classifications” (Müller Staub et al., 2015, p. 13). This *Advanced Nursing Process* is an advancement of the traditional nursing process and bases on the body of knowledge, which includes the internationally recognized nursing diagnosis classification of NANDA‐I (Müller Staub et al., 2015). As the risk for malnutrition is missing (Herdman & Kamitsuru, 2018), it is impossible to implement the *Advanced Nursing Process* regarding this phenomenon. Hence, the presented research project focused on developing a standardized nursing diagnosis for the risk for malnutrition, classified according to NANDA‐I, which is the most often used classification in scholarly books and implemented in electronic health record systems worldwide (D'Agostino et al., 2019; Jones et al., 2010; Tastan et al., 2014). Nursing diagnoses are clinical judgements about current or potential reactions to the health problems of individuals, families or communities. They are the basis for the choice of nursing interventions to achieve nursing outcomes for which the nurse is responsible (Müller Staub et al., 2015, 2017).

**FIGURE 1:**
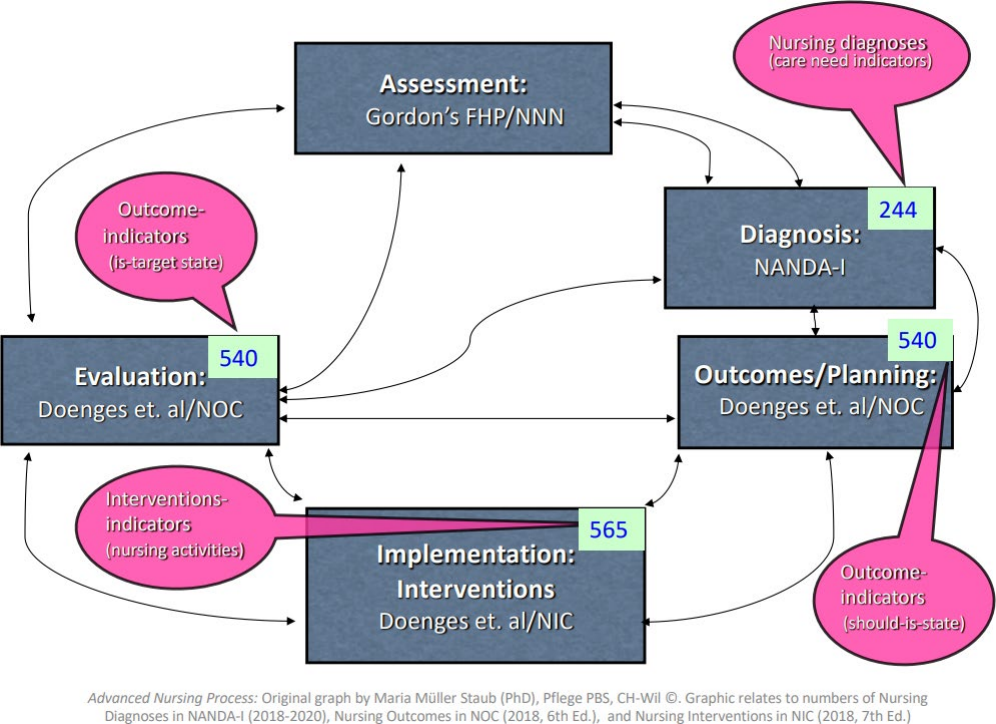
Advanced Nursing Process (Müller‐Staub, 2020)

**FIGURE 2:**
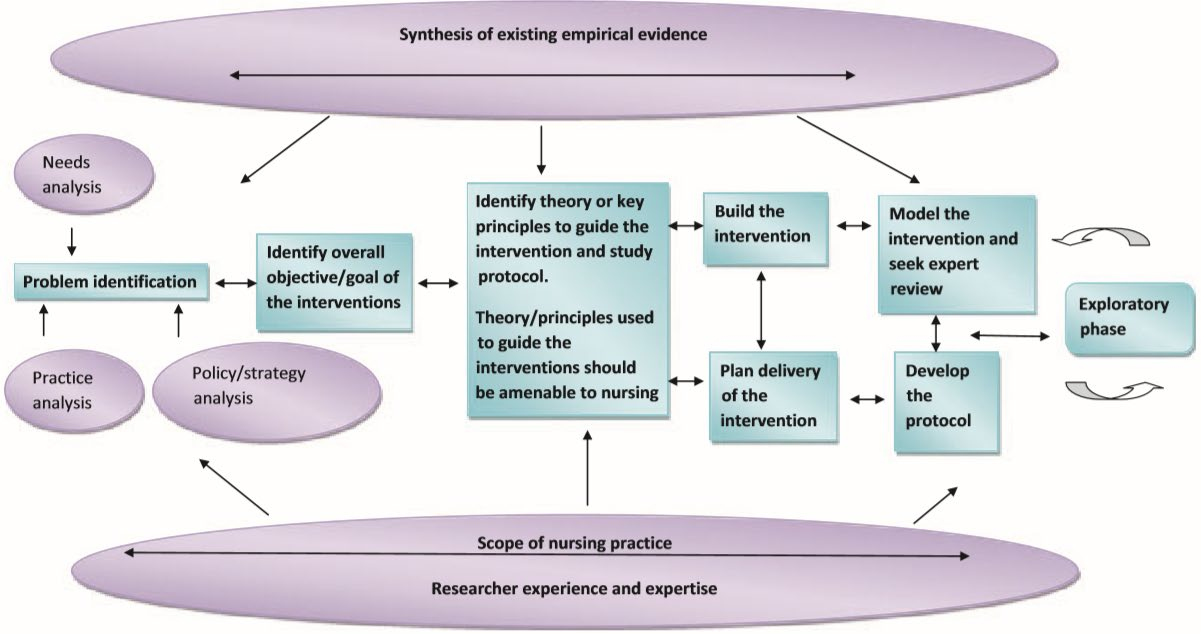
Model for developing complex interventions in nursing (Corry et al., 2013)

Risk nursing diagnoses are formulated with labels, definitions (problem descriptions) and risk factors, which are subdivided into: (a) risk factors; (b) at‐risk population; and (c) associated conditions (Herdman & Kamitsuru, 2018). Nurses need to be aware of at‐risk populations and associated conditions, as these may increase the risk for malnutrition while not being directly controllable by nurses. What is more, nurses apply the concepts described in the classifications through clinical decision‐making. This clinical reasoning requires a high level of expertise and strong clinical decision‐making skills, as shown by Müller‐Staub's model (Figure 1). Corry et al. (2013) established a model for developing a complex nursing intervention that allowed a structured and comprehensible mixed‐methods approach, considering the nurses' and hospitals' context (Figure 2).

Identified theory or key principles could be a risk nursing diagnosis according to the standards of NANDA‐I. Thus, a nursing diagnosis describing the risk for malnutrition is elaborated. In the presented part of the research project, we focused on developing a risk nursing diagnosis, congruent with steps (1) and (3) of Corry et al.'s model. Step (1) is described as precise identification of the problem based on three fields: Needs analysis, practice analysis and policy analysis that entails examining current literature, including the locally applicable standards and guidelines for action. Step (3) is defined as identifying key risk factors and linking them to appropriate labels and definitions for NANDA‐I nursing diagnoses (Table S1). Nurses must be aware of patients' risks and enabled to name what they see, which is also valuable in describing risk factors (Clark, 1999).

### 1.2 Purpose statement

The purpose of this mixed‐methods study was to develop a nursing diagnosis defining the risk for malnutrition. The following research questions were answered:

- How can an evidence‐based risk nursing diagnosis concerning malnutrition in older people in hospitals be formulated regarding label, definition and risk factors?
- Which of the identified risk factors are substantiated by empirical data regarding patients' perspectives (QUAL), practice observations (QUAL) and participants' nursing records (QUAN)?

## 2 THE STUDY

### 2.1 Design

**FIGURE 3:**
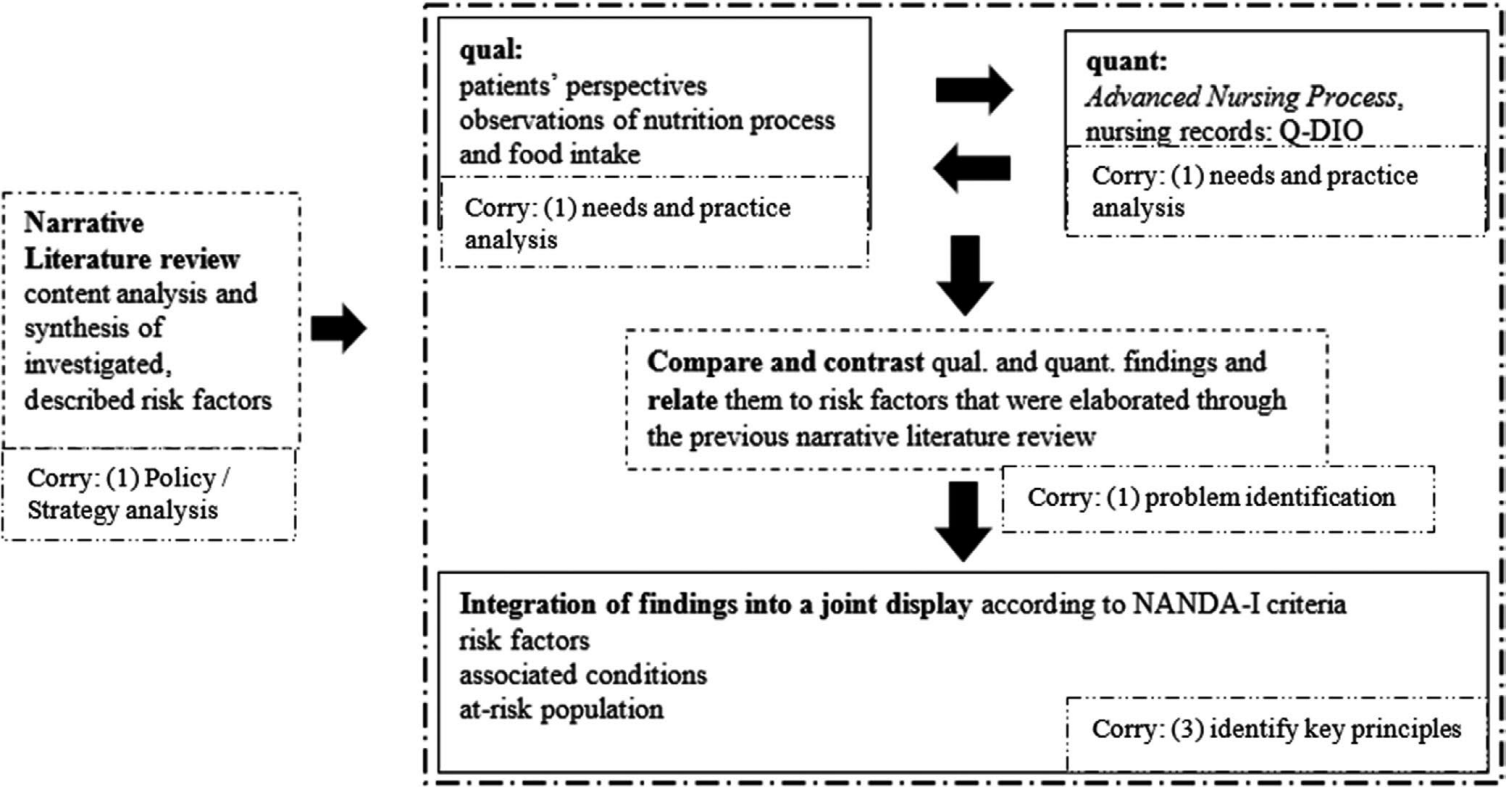
Convergent parallel mixed‐methods design (Figure elaborated by the authors)

A narrative review of the literature built the basis of evidence and structure of label, definition and risk factors of a risk nursing diagnosis (Figure 2, Model of Corry et al., 2013). Design, setting and sampling of each step are described in the methods section. Several methodological approaches were taken to define the risk and risk factors for malnutrition in older people in hospitals. A convergent parallel mixed method design was applied to capture qualitative ontological points of view and multiple social realities (Hesse‐Biber & Johnson, 2015). This mixed method design aimed to understand and integrate various subjectivities (Creswell, 2015). Therefore, qualitative and quantitative data have been collected independently of each other's content and analysed during the same period (Creswell & Plano Clark, 2018). Individuals were seen as experts in social constructionism's philosophical stance (Berger & Luckmann, 2011). In this sense, patients could tell their nutritional problems and express their nutritional needs leading to risk factors for malnutrition. Therefore, qualitative interviews, observations of patients and nursing record analyses were conducted. These three data collection methods supported seeking internal validity and credibility of the new nursing diagnosis, as suggested by Miles et al. (2014). The procedure of this convergent parallel mixed method design is displayed in Figure 3.

### 2.2 Methods and analyses

The convergent parallel mixed method design meant that qualitative interview and observation data for the needs and practice analyses were collected during 1 day for each participant (Creswell & Plano Clark, 2018). Quantitative data from nursing records were collected after the participants' discharge. The data collection methods, analysis and data integration of the different steps of this mixed method design are presented in the subsequent sections.

#### 2.2.1 Literature review (qualitative data)

A specific search was conducted in CINAHL and PubMed according to current nursing theories of literature search (Holly et al., 2017; Kleibel & Mayer, 2011) to identify an evidence‐based label, a definition and the risk factors. Two independent reviewers performed searches in October 2018, using the following MeSH terms: “aged, 80 and over” and humans, or “inpatients/organization and administration” or “inpatients/rehabilitation” or “inpatients/statistics and numerical data” or “humans and nutritional status” or “nutrition assessment” or “nutrition surveys” or “diet, food and nutrition” or “nutritional, physiological phenomena” and humans. The selection of studies (inclusion [in]) and exclusion [ex]) was performed following the PICOTSS framework (Kleibel & Mayer, 2011) (Table 1).

**TABLE 1:** PICOTSS format inclusion and exclusion for studies of the narrative literature review

	Inclusion	Exclusion
**P**opulation	Older people, aged 80 years and above	Young adults, children, terminally ill, specific disease (e.g. focus on specific cancer patients)
**I**nvestigated subject	Risk factors for protein–energy malnutrition	Intervention‐testing
**C**ontext	Any country	Not applicable
**O**utcome	Identified risk factors or associated conditions on the nutritional status, amount of food intake, nutritional status according to an assessment tool (e.g. Mini Nutritional Assessment [MNA]), weight, functional status (e.g. handgrip)	Validation of screening tools, comparison between screening tools
**T**ime	Any, no limit of publication dates (beginning until 08/2019)	None
**S**etting	In hospital OR institutions	Community setting and ambulatory care, intensive care unit, palliative care unit
**S**tudy type	Peer‐reviewed, abstracts in English, and full text in English or German	Non‐peer‐reviewed articles, expert opinions

According to tools from the Joanna Briggs Institute, the literature synthesis involved a critical appraisal depending on the included studies' design (Aromataris et al., 2017). This critical evaluation of previous studies helped the researchers to assess the results. Investigated risk factors of the narrative literature review were merged as risk‐factor‐building codes in a table following the structure of summarizing inductive content analysis. This content analysis included paraphrasing, generalizing and reducing to reach a consistent level of abstraction (Mayring, 2015), as a crucial step in developing a nursing diagnosis.

#### 2.2.2 Context, observations and interviews (qualitative data)

One ward per clinic (perioperative, internal medicine and acute geriatric care) of a 200‐bed Swiss hospital was involved. This purposive sampling aimed to include a similar number of participants from each ward (*N* = 6–8), also known as sampling for range (Small, 2009). Older people were informed about the research project and asked to participate via verbal and written information communicated by the project leader 1 day before data collection if they were as follows: (a) aged 80 years and above; (b) staying in the hospital for at least 5 days; and (c) able to sustain a dialogue in the German language. In case of: (a) being terminally ill; (b) being tube‐fed; or (c) receiving parenteral nutrition, they were excluded. Patients' relatives were asked to participate in the interview if patients were cognitively impaired, as stated by a medical diagnosis or nurses' report. Data collection took place on predetermined days (1 day per participant) from September–November 2018. Three Advanced Practice Nurses (APNs), one from each of the three clinics, four research assistants and one nutritionist, were part of the research team to observe the patients' feeding and nutrition processes in the hospital. They collected the patients' views of these procedures and their nutrition status. Observation days started at 07:30 a.m. and entailed breakfast, lunch and dinner (6:30 p.m.). The semi‐structured 40‐item observation guide entailed tasks such as feeding support, hygienic measures before meals (i.e. washing of hands) and discussions of nutrition topics between participants and nurses during ward rounds (Appendix S1). The observers stayed on the ward, wearing hospital‐staff clothing, not to attract attention during the daily care routine. They took notes during or immediately after observation sequences, for example, after plates were served or after the nurses' shift‐hand‐over in the afternoon. Data were analysed as observations documented in the observation guide and summarized by re‐writing and re‐arranging the texts into a concise description (Bethmann, 2019).

Perspectives of older people were recorded at their bedsides using a semi‐structured interview schedule with ten topics and three questions regarding the participants' characteristics and demographic information (Appendix S2). The participants were asked about their appetite, breakfast preferences and whether the food intake was evaluated properly. The interviews were transcribed verbatim according to simple transcription rules (Dresing & Pehl, 2018). Following this, observation data and interview transcripts were encoded deductively according to the risk‐factor‐building codes from the literature review using the qualitative data analysing software ATLAS.Ti 8 (ATLAS.ti, 2019). Intercoder agreement about the level of abstraction was sought and achieved through discussion within the research team.

#### 2.2.3 *Advanced Nursing Process*, nursing records (quantitative data)

The quality and internal coherence of the *Advanced Nursing Process* entailing the accuracy of nursing diagnoses, the effectiveness of related nursing interventions and the quality of nursing‐sensitive patient outcomes were quantitatively evaluated with the validated instrument Quality of nursing Diagnoses, Interventions and Outcomes (Q‐DIO) (Müller‐Staub et al., 2009). The evaluative, descriptive analysis of the older people's nursing records (from the interviewed and observed participants) was accomplished using the Q‐DIO‐Nutrition (Q‐DIO‐N). The Q‐DIO‐N has been adapted with the permission of the instrument developer M. Müller‐Staub ( Personal communication) from the instrument Q‐DIO (Müller‐Staub et al., 2008, 2010). The Q‐DIO‐N focuses on nursing diagnoses related to nutrition. It contains 27 items. Each item rated on a three‐point Likert‐type scale with the following response options: 0 = not documented, 1 = one aspect is documented with medium quality, 2 = two or more aspects are comprehensively recorded in good quality (Table S2). The accuracy of nursing diagnoses relies on the format with problem definition, aetiological or risk factors and symptoms (defining characteristics), leading to interventions and nurse‐sensitive patient outcomes (Johnson et al., 2011; Müller‐Staub et al., 2009). Coherence implies that accurately formulated nursing diagnoses are correctly linked with effective nursing interventions and related outcomes. The four dimensions measured with the Q‐DIO‐N were as follows: (a) quality of the nursing diagnosis as a process, namely assessment and diagnosis selection; (b) quality of the nursing diagnosis as a product, addressing title and definition, defining characteristics and aetiological or risk factors; (c) quality and effectiveness of nursing interventions; and (d) nursing‐sensitive patient outcomes (Müller‐Staub et al., 2008). Consequently, correlations between Q‐DIO‐N scores were calculated with SPSS Version 25 (IBM, 2019). The level of significance was set at *p *< .05; Pearson's correlation was assumed as strong at *r* = 0.5, medium at *r* = 0.3 and weak at *r* = 0.1 (Cohen, 1988, p. 285ff). Special details were entered as memos (very precise and accurate documentation). Additional data, such as age, length of stay, number of medications, number of medical and nursing diagnoses and the nutrition risk score (NRS‐2002) at admission were obtained. Regarding psychometric properties, the inter‐rater reliability of Q‐DIO‐N showed a Kappa of 0.70 and the intra‐rater reliability was 0.82 when two researchers independently analysed seven nursing records (189 items). The mean values per item dimension's sum scores were calculated, and correlations were sought between the four dimensions of the *Advanced Nursing Process*.

#### 2.2.4 Data integration

Risk factors elaborated from the narrative literature review built the code‐matrix and, therefore, the basis of a joint display explained by mixed method researchers (Creswell & Plano Clark, 2018; Rädiker & Kuckartz, 2019). A joint display showed integrated data from risk factors, indicators, hints or shortened stories (Creswell & Plano Clark, 2018). This mixed data from interviews, observations and descriptively analysed and interpreted quantitative data from nursing records yielded enriched and contextually validated risk factors to seek credibility (Creswell & Plano Clark, 2018; Teddlie & Tashakkori, 2006). Data integration was a non‐linear approach. Each data corpus (interview transcripts, observation notes, Q‐DIO table) was analysed separately by the first author and research assistants (Master's students). The elaborated models of risk factors and integrated findings were discussed monthly with the supervisors. Based on these findings and requirements of standardized nursing language (SNL), the label, definition and risk factors for malnutrition were formulated. The integration of different data and different points of view from the literature, including interviews with older people in hospitals, observations and nursing records, was performed at the stage of data synthesis and led to a reliable risk nursing diagnosis to define the risk for malnutrition in older people in hospital.

#### 2.2.5 Rigour, validity and inference quality of the mixed method study

In this mixed method study, high quality and scientific rigour have been attained through the transparency of the methodology and the accuracy in the description of data integration, as proposed by Collins (2015). The research group elaborated observation and interview guides and tested them for applicability and content validity by performing a pre‐test with four APNs. This pre‐test's findings were discussed within the research team on two occasions and minor adjustments were made, as suggested by Polit and Beck (2017). To ensure the quality and consistency of the qualitative data collection, each member of the research group performed a pilot investigation under the project leader's supervision lasting at least half a day. These research tasks enhanced the credibility and trustworthiness of qualitative data. The research group discussed the findings regularly and gave the first author feedback to reduce the risk of overseeing important aspects. This whole process was supervised by a senior researcher.

This study's validity can be described as inference quality, which entails the full description of risk factors by integrating the multiple perspectives of older persons, practice observations and nursing records (Collins, 2015; Polit & Beck, 2017). Several feedback rounds in the research group and from experienced mixed method research supervisors afforded the authenticity of qualitative data and the correctness of quantitative data and data integration. The development of risk factors was critically discussed within these groups. Credibility was sought as the authors reviewed the findings in academic societies collaborating on SNL and nursing diagnoses classifications. A bilingual nursing researcher and member of the Swiss Association for Nursing Science translated patient interviews and observation records.

### 2.3 Ethics and research reporting checklist

The study focused on facilitating the theoretical knowledge of the nursing practice. It was approved by the regional ethical review board (Req‐2016‐00670), by the University of Vienna and the local institutional board for quality management. A frequently neglected group of people (older people with cognitive impairment, such as dementia) was included by involving representatives, such as family members. Participants or their legal representatives received verbal and written information about the study's aims and their right to quit participation anytime. They signed a consent form and were given a pseudonym as soon as data were entered in a data collection file. Any documents were stored in a locked sideboard while digital data were stored on a password‐secured computer. The present study was self‐evaluated using the GRAMMS criteria (Cameron et al., 2013).

## 3 FINDINGS

First, the risk nursing diagnosis is presented, followed by a description of the 22 participants' characteristics. An overall summary of the quantitative findings of the nursing records is displayed afterwards. The synthesis of the literature review and integrated data from three exemplary cases (interviews, observations and nursing records) that indicated the risk factors and described them with their meaning in more detail (content validation) is presented in a joint display (Table 2).

**TABLE 2:** Joint display of integrated findings of risk factors for malnutrition elaborated by the findings of the literature and supported by empirical evidence from nursing records (QUAN), interviews (QUAL), and observations (QUAL), three exemplary cases (Ms. P. with highest Q‐DIO‐N sum scores, Ms. O. with medium scores, and Ms. R. with lowest Q‐DIO‐N scores)

Risk factors (References from Literature review)	Exemplary case: best Q‐DIO value (Ms. P., geriatric care ward)	Exemplary case: medium/mean Q‐DIO value (Ms. O., perioperative care ward)	Exemplary case: worst Q‐DIO value (Ms. R., internal medicine)
**Health care workers' attitude, culture – missing awareness** Lindorff‐Larsen (2007) Peng et al., (2015) Volkert et al. (2010)	The nursing record of Ms. P. demonstrated impressively the effect of nurses' awareness: After setting the nursing diagnosis, the older person's appetite and the suggestions of the speech therapist were recorded and finally Ms. P. reached an intake of > 100% of her protein and energy requirements. Reports between nurse and doctor entailed digestive problems; the service team could ask the nutritionist about garlic intolerance or allergy. Nevertheless, the consumed amount of food was not correctly documented (fruit juice was taken, while documented as “not‐taken,” and at lunch, the potatoes were checked off as completely eaten, while there were still three tablespoons of potatoes left).	Ms. O. expressed that she received a semi‐portion and felt hungry afterwards. Nobody explained it to her that it was up to her to choose the menu size. She explained that it depended on the individual nurse assistant whether coffee was poured or not. She had one forearm in a cast and could not use the other arm due to shoulder pain. Ms. O. realized the difference between observation‐day and the other days, when no one actively asked her about her appetite.	Reduced intake and soft‐textured food were recorded in the daily care routine documentation system, without a problem‐focused nursing diagnosis malnutrition (no aims, no specific interventions planned), which indicated a lack of prioritizing food support and a lack of nutrition enhancing culture (Q‐DIO remarks, Ms. R.) ‐ lowest Q‐DIO total scores. This is in congruence with the observations: Ms. R. had pain, did not get dentures before breakfast, was sitting uneasily with the consequence that she did not eat a lot but ate very quickly instead ‐ in order not to need to sit for a long time in a painful position. The information about pain was not handed over from one nurse to the next. It can be dangerous or at least unpleasant, and NOT appetite‐enhancing if patients do not get their preferred food in the needed consistency. One reason might be that it is not team culture to involve the patients' relatives and ask about the patients' eating habits, or about the quantity of food consumed at home ‐ as heard and observed in the cases of Ms. R., Mr. S. (intern medicine), and Ms. J. (geriatric care ward).
**Inappropriate mealtime environment** Nieuwenhuizen, Weenen, Rigby, Hetherington, (2010) Patel & Martin, (2008) Rubenstein et al., (2001) Söderström et al., (2013)	Ms. P. was well and comfortably seated (got pain medication 10 min before getting up for breakfast, feet on the ground). The room was dark, as her eyes were very photosensitive. Serving dinner, the nurse assistant used motivational prompts “Oh, it looks delicious, enjoy your meal!” The inappropriateness was the mealtime disturbance, as the registered nurse‐delivered medication during mealtimes.	As Ms. O. explained during the interview, it was a matter of chance whether the napkin and glass of drink were in place for the meals or she had to ask for them. There was fresh air in this room during mealtimes, Ms. O. could refresh her hands before dinner on the observational day.	Relatives (son and husband) were not involved but showed interest in knowing where Ms. R. was eating (at the table or bedside?) during the interview.
**Impaired oral cavity status (oral health)** Chen (2007) Chen, Tang, Wang, Huang, (2009) Hasseler (2010) Nieuwenhuizen et al., (2010) Patel & Martin, (2008)	Dentures were in the mouth during the night. No support was offered to rinse the mouth preventing mucositis in the morning nor before any other meal (observation Ms. P.). Ms. P. explained that she could only call for help, for example, to go to the bathroom for brushing her teeth anytime.	Ms. O. had her own teeth. She explained that she did not find time before breakfast to rinse her mouth, but afterwards.	Ms. R. realized that dentures were missing when she had started to eat. As dentition on the lower jaw did not fit well, she had to get pureed food, which was not ordered before the day of observation. During the interview, it became clear that chewing was impossible with only one proper tooth.
**Impaired swallowing** Mudge, Ross, Young, Isenring, Banks, (2011) Namasivayam (2015) Patel & Martin, (2008) Pirlich et al., (2005)	The service staff member who ordered food admitted not knowing whether a patient had difficulties in swallowing or not; this was not written in his records. According to the patient's record (documentation), Ms. P.'s main diagnosis was stroke; swallowing ability was tested and observed by speech therapist, as described in the nursing record.	Problem with swallowing denied during interview and not observed either.	Ms. R. took food in her mouth, was not able to swallow, often spit it out again, and therefore she lost weight, as the husband told, helplessly.
**Polypharmacy and/or multimorbidity** Bonetti et al. (2017) Chen (2007) Chen et al., (2009) Müller Staub et al. (2017) Pirlich (2005), Rubenstein et al., (2001	Medication or multimorbidity was not a topic during the interview or observation.	Although Ms. O. had severe renal insufficiency with limited liquid intake, the amount of water, tea or soup was not documented by the nurses. The service team had ordered soup despite drinking restrictions.	Ms. R. suffered from diabetes, renal insufficiency with weight gain due to oedema. The diabetes counsellor came to the ward without talking to Ms. R. or to her husband (observation and interview).
**Appetite loss** Mudge et al., (2011) Nieuwenhuizen et al., (2010) Peng et al., (2015) Rubenstein et al., (2001 Volkert et al. (2010)	Ms. P. had emesis. The nurse supported her to rinse the mouth. There was no inquiry whether she might want to eat something later in the evening, but the dinner was brought away (observation). Ms. P. was convinced that the staff checked how much she ate and that they would recognize if she did not eat enough. (interview). In Ms. P.'s point of view, it seemed normal that her appetite varied, in the hospital as at home.	Ms. O. disclosed never being asked about her appetite. She felt hungry before breakfast and dinner (interview). Sometimes she did not feel like having meat.	Until the day of the observation, there was no nursing diagnosis related to food intake or appetite. The nurse assistant helped her getting back to bed after lunch, and asked whether she had had enough; Ms.R. expressed not having an appetite (observation). After two spoons of soup, or two bites ‘it was closed’; then she could not eat anymore. The reasons for that were unclear until the day of the observation.
**Care dependency** Bonetti et al. (2017) Chen (2007) Chen, Dai, Yen, Huang, Wang, (2010) Jacobsen (2016) Mudge et al., (2011) Nieuwenhuizen et al., (2010) Peng et al., (2015) Pirlich (2005) Rubenstein et al., (2001 Schrader (2014) Söderström et al., (2013)	Ms. P. Was not recorded as needing help for eating, although she got help by her relative as she was too weak and visually impaired to eat on her own (observation, nursing record). She got analgesia before breakfast and got aid to wash her face and rinse her mouth after vomiting.	Ms. O. got help to eat her meals; the nurse assistant who brought food asked her whether she should put butter on the bread. Ms. O. asked for help to pour milk and coffee. She expressed dependency on the staff in terms of the portion size, as she did not dare to ask why she only got half a serving.	There was no nursing diagnosis related to food intake or impaired oral cavity status, nor for malnutrition, which would be needed according to nursing records, interview, and observation data. Ms. R. got her dentures fixed during breakfast—without asking how she is places them. The nurse assistant automatically put adhesive paste on, which was uncomfortable for Ms. R.

### 3.1 Narrative review of the literature

**FIGURE 4:**
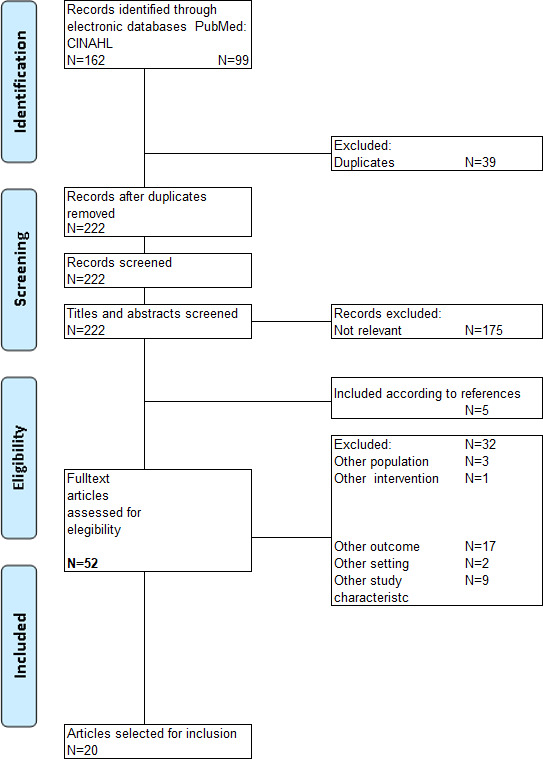
Flow diagram according to the PRISMA statement (Moher et al., 2009)

Twenty studies from ten different countries published between 2001–2017, describing risk factors for malnutrition in older people, were included (Figure 4). The literature search generated three reviews and 17 pre–post‐test, observational, cross‐sectional and exploratory studies with low to high risk of bias (Tables S7‐S10). Summary tables of the included studies with codes for risk factors of malnutrition are displayed in Tables S5 and Table S6.

### 3.2 Risk nursing diagnosis

Label and definition were determined according to the European Society for Clinical Nutrition and Metabolism (ESPEN) (Volkert et al., 2019) and set in congruence with the terminology of NANDA‐I (Herdman & Kamitsuru, 2018).

**Label.** The risk for protein–energy malnutrition.

**Definition.** Susceptible to inadequate coverage of estimated protein–energy needs due to risk factors, including acute or chronic illness and advanced age.

The risk for protein–energy malnutrition in older people has been defined by a nutritionists' expert consensus as follows:

Older persons are at risk for malnutrition if oral intake is markedly reduced (e.g. below 50% of requirements for more than three days) or if risk factors, which either may reduce dietary intake or increase requirements (e.g. acute disease, neuropsychological problems, immobility, chewing problems, swallowing problems), are present. (Volkert et al., 2019, p. 11)

**Risk factors.** The following risk factors were identified:

- Healthcare workers' attitude and culture—missing awareness
- Inappropriate mealtime environment
- Impaired oral cavity status
- Impaired swallowing
- Polypharmacy and multimorbidity
- Appetite loss
- Care dependency

**At‐risk population:**

- Age (65 years and above)
- Low socioeconomic status
- Female gender.

**Associated conditions:**

- Acute disease
- Body composition (enhanced requirements)
- Neurocognitive disorder (dementia)
- Sensory decline
- Weight loss in the last 3 months
- Psychiatric factors (depression)
- Social isolation.

### 3.3 Synthesis of the mixed‐methods study

#### 3.3.1 Participant characteristics

Thirty‐seven patients were asked to participate on the predefined dates; 15 refused to take part for the following reasons: too ill (3), perceived themselves disabled to meaningfully contribute (3), declined to participate (8), or were discharged unexpectedly (1). The mean age of the 22 included participants was 86.1 years (range = 80–94 years); 16 were females and six males and the length of stay ranged from 5–30 days (median 12 days). The participants lived on their own (*N* = 19) or in a long‐term care institution (*N* = 3). One of them had to care for a relative, seven cared for their household and four looked after plants, while ten participants did not care for anything. The total observation time was 198 hr, with a mean observation period per patient of 9 hr.

#### 3.3.2 Quantitative findings of nursing records

The *Advanced Nursing Process* of 22 evaluated health records included the following nursing diagnosis (number in parentheses): imbalanced nutrition—less than body requirement (10), risk for constipation (1), constipation (1), feeding self‐care deficit (1) and no nutrition‐related nursing diagnosis (9). On average, a nutrition‐related nursing diagnosis was set on the fourth day after hospital admission and correlated positively with the NRS‐2002. The highest sum square score was reached in Q‐DIO‐N as a process (1.34, range: 0–2). The mean value of the Q‐DIO‐N outcome (evaluation of the planned care) demonstrated the lowest score with 0.23 (range: 0–2) as displayed in Table S3 and Table S4. A significant correlation between Q‐DIO‐N product and Q‐DIO‐N outcome scores was registered (Pearson's rho = −0.407, *p *= .03). The correlation between Q‐DIO‐N scores of the nursing diagnosis as a process (assessment) and as a product (title, defining characteristics and aetiology criteria) was not significant (Pearson's rho = 0.242, *p *= .278). No significant correlation between the Q‐DIO‐N score as a process and as an intervention was found (Pearson's rho = 0.262, *p *= .238). The nursing diagnoses were rarely evaluated concerning the planned nursing interventions' objectives or effectiveness, which might have caused the lack of correlation between Q‐DIO‐N scores as a process and as an outcome (Pearson's rho = 0.219, *p *= .372). Six participants had a high risk without any prescribed nutrition supplements. The joint display (Table 2) presents one case per clinic from the multi‐case analysis with extreme cases and one average participant selected according to the Q‐DIO‐N scores. The exploration of Ms. P. demonstrated the highest Q‐DIO‐N scores. Ms. O. met the criterion of being closest to the median of the sum scores per Q‐DIO‐N item dimension. In contrast, the case of Ms. R. was selected as it demonstrated the lowest Q‐DIO‐N scores.

#### 3.3.3 Detailed description of risk factors

Integrated findings with specific meanings due to examples of interviews, observations and nursing records analysis are described in the following sections for each risk factor. “Health care workers' attitudes and culture – missing awareness” for nutrition and eating as part of the therapy is a risk for malnutrition in older people in hospitals. The missing awareness for the importance of between‐meal snacks was shown when a nurse assistant served a protein‐enriched cream together with the main meal at lunchtime, which was removed with the meal tray 40 min later. Furthermore, liquid intake most often was not recorded, despite cardiac or renal diseases. Relatives were not involved in assessing food preferences or nutrition therapy, which seemed to be part of the nursing team culture.

“Inappropriate mealtime environment” is a risk factor that stands for the surrounding of food intake, including organizational factors, such as time between meals, overnight fasting periods or mealtime disturbances. A quarter of all meals were eaten at the bedside, without any social support. Another aspect was the missed opportunity to refresh oneself before meals (e.g. washing hands and rinsing the mouth). “Impaired oral cavity status” is a further risk factor observed in three participants who talked about loose or lacking dentures owing to which they could not eat all kinds of food. Therefore, they left out vegetables or meat.

“Impaired swallowing” is a risk factor, which was difficult to recognize, as seen in the following example: In one situation, it was observed that the doctor asked for dysphagia due to Parkinson's disease on his ward round. At the same time, the participant was not supervised but left alone during mealtime. The husband of one participant said during the interview: "… pureed (food), yes, so that she can swallow it" (husband of Ms. J.).

“Polypharmacy and multimorbidity” were often not taken into consideration by the health care staff. One participant reflected that her perception of taste altered in the hospital: "It had a strange metallic taste, the meat and all the food had that same strange taste, (…) Maybe it is because of the medication." The 22 participants had 8.5 (median) prescribed medications per day.

“Appetite loss” was rarely assessed. However, many participants were affected by appetite loss, as seen in the following examples: "Generally, I lost appetite in my advanced age." Another participant confirmed: "I eat because I have to eat, not for joy."

“Care dependency” is a risk factor, as many older people in the hospital cannot eat independently due to impaired mobility, cognitive decline or sensory loss (visual or hearing impairment). As one participant confirmed: "At the moment I cannot use the knife because of this cast on the forearm, sometimes I get help."

## 4 DISCUSSION

The aim of the study has been achieved: An evidence‐based risk nursing diagnosis with label, definition and risk factors concerning malnutrition in older people in hospitals is presented. This new nursing diagnosis offers 18 risk factors, including associated conditions and characteristics of the at‐risk population. The mixed method design fostered the determination of the risk factors by enabling a context‐related empirical content validation of the phenomenon *risk for protein–energy malnutrition in older people in hospitals*. The mixed method design helped to verify and strengthen the risk factors found in the literature review, as there is no rule for how to weigh the different findings and components of published evidence (Holly et al., 2017). According to the NANDA‐I classification, the level of evidence of this clinically supported risk nursing diagnosis would be 3.2 “Clinical studies related to diagnosis, but not generalizable to the population” (Herdman & Kamitsuru, 2018, p. 6). Label, definition and risk factors were literature‐based, Medical subject Headings were displayed, and patients were subjects for content and context validation of risk factors. Furthermore, expert opinion was sought and integrated into the process of defining the risk factors and data integration.

Research supports that SNL—mainly nursing diagnoses—enhance nursing‐sensitive patient outcomes (Leoni‐Scheiber et al., 2020; Müller‐Staub et al., 2006; Müller‐Staub et al., 2008; Watson et al., 2014). The developed nursing diagnosis supports nurses' clinical judgement to prioritize preventive nutritional care. The nursing records of two participants also showed direct benefits of SNL, as after setting a nursing diagnosis related to nutrition, the intake of protein drinks and feedbacks to nutritionists were documented more precisely and thoroughly. The risk factors in the context of SNL and their potential interrelationships are discussed below.

Nurses' attitudes towards older people's care could be affected by a lack of knowledge in caring for older people (Hanson, 2014). Therefore, it is imminent that the risk for protein–energy malnutrition is defined as part of the SNL, thus part of the body of knowledge in nursing. The elaborated risk factors are now made explicit and can become part of the NANDA‐I nursing diagnoses classification. The need for a risk nursing diagnosis is supported by nutrition experts who have stated that screening the risk and realizing the problem is the first step in preventing malnutrition (Soeters et al., 2017).

As stated in the introduction of this article, some nutrition‐related nursing diagnoses exist within the NANDA‐I classification. An example is “impaired swallowing.” This diagnosis includes protein–energy malnutrition as an associated condition (Herdman & Kamitsuru, 2018, p. 173), which seems to be a vicious circle, if unrecognized. Another related nursing diagnosis is “feeding self‐care deficit,” within which “environmental barrier” is a related factor and one of the defining characteristics is the “impaired ability to swallow food” (Herdman & Kamitsuru, 2018, p. 245). “Feeding self‐care deficit” is in congruence with the elaborated risk factor “care dependency.” These findings demonstrate that nursing diagnoses influence each other. As observed in practice, one reason for appetite loss was suffering from nausea and emesis. Two participants mentioned nausea; sometimes, they got antiemetic drugs, sometimes there was no documented intervention, which might be a problem of healthcare workers' attitude, as treating nausea did not seem to be a priority. These potential interrelationships of various risk factors and existing NANDA‐I nursing diagnoses allow us to think of a syndrome nursing diagnosis, which is defined as a cluster of two or more nursing diagnoses that occur together (Herdman & Kamitsuru, 2018). Therefore, to investigate the extent to which several risk factors or nursing diagnoses are often present in combination and might be addressed together could be the subject of further research. The associated condition of social isolation indicates the necessity to provide empathetic support or to arrange spiritual or psychological assistance. This in turn might increase nutrition intake, as one participant expressed: "I force myself to eat (…) I was caring for my husband for many years until he died.”

Even though the present study was restricted to in‐hospital older people, previous studies in primary care settings support the current findings. Håkonsen et al. (2019) defined a Minimum Data Set for Nutrition that included: (a) physiologic measurements; (b) ability to eat; (c) intake; (d) stress factors; and (e) factors which indirectly affect older people's protein‐ and energy‐intake and needs. The literature supports that a standardized language empowers healthcare professionals, as "communicating nutrition‐related observations with relevant stakeholders, such as nurses, dieticians, general practitioners and the management," is of primary importance (Håkonsen et al., 2019, p. 11). It is within the domain of nursing to assess appetite, weight loss and eating behaviours (Mirmiran et al., 2011).

### 4.1 Strengths and limitations

This research is the first describing a nursing diagnosis *risk for protein–energy malnutrition,* including older people's and their relatives' points of view. In previous research, the perspectives of older people with cognitive impairment and of their relatives were missing. A limitation of this study is that there was no systematically driven expert review, such as a Delphi study.

## 5 CONCLUSION

The new nursing diagnosis “Risk for protein–energy malnutrition” makes a vital contribution to the body of knowledge in SNLs. It empowers nurses to describe older people's nutritional needs in the interdisciplinary team. Further, this piece of SNL makes nursing visible in society, for education and towards policymaking. Recognizing the risk for protein–energy malnutrition will help nurses to prevent malnutrition and its associated complications. It is highly recommended to raise nurses' awareness of this vital topic by implementing this diagnosis into clinical practice. Further research is needed to clinically test and validate this risk nursing diagnosis in multiple healthcare facilities.

## CONFLICT OF INTEREST

All authors declare no conflict of interest.

## Supporting information

Supplementary Material

## Data Availability

The data that support the findings of this study are available in the supplementary material of this article.

## APPENDIX 1

### GRAMMS Good Reporting of a mixed‐methods study

**Table:**

Criteria A scoring system for mixed methods research and mixed studies reviews. Types of mixed methods study components or primary studies in a SMSR context Methodological quality criteria	Yes, fulfilled, page number	not applicable/not enough information
1. Qualitative		
_ Qualitative objective or question _ Appropriate qualitative approach or design or method _ Description of the context _ Description of participants and justification of sampling	4 5–10 6 (context, observation…) 6–7	
_ Description of qualitative data collection and analysis _ Discussion of researchers' reflexivity	6–7 9–10 (rigor, inference validity)	
2. Quantitative experimental		
_ Appropriate sequence generation and/or randomization _ Allocation concealment and/or blinding _ Complete outcome data and/or low withdrawal/drop‐out		X X X
3. Quantitative observational		
_ Appropriate sampling and sample _ Justification of measurements (validity and standards) _ Control of confounding variables	6–7 (observations, interview) 9–10 (psychometric properties, rigor, validity)	X
4. Mixed methods		
_ Justification of the mixed methods design _ Combination of qualitative and quantitative data collection‐analysis techniques or procedures _ Integration of qualitative and quantitative data or results	5 (Design) 5–9 (Qual. and Quan. data collection) 9 (data integration)	

Caution notice: Outside quantitative experimental studies, the implication of clustering primary studies or study components by quality score has not been critically examined. With respect to systematic reviews of quantitative experimental studies, the clustering of primary studies and the weighting of quantitative results by quality score are discouraged.

a Potential applications: With respect to mixed methods research in general: Appraisal of the methodological quality of qualitative, quantitative and mixed methods components. With respect to systematic mixed studies reviews: Concomitant appraisal of the methodological quality of primary qualitative, quantitative and mixed methods studies.

b Procedure for planning, reporting and assessing mixed methods research or mixed studies reviews. For each type of study component or primary study, describe the methodological quality by criterion. Score presence/absence of criteria, respectively 1/0 (complement the retained publication with related documents, and contact authors when more information is needed). Calculate a “quality score” [(number of “presence” responses divided by the number of “relevant criteria”) _ 100]. Use this score as a rationale for excluding “poor quality” study components or primary studies. Use the criteria for describing the quality of retained components or studies (qualitative quality appraisal) (Pluye et al., 2009).

**Table:**

**Assessment of the success of MM studies** (O'Cathain et al., 2008)	**Yes (y)**, fulfilled, described on page (p.)	**no**	**not applicable/not enough information**
1 Is the quantitative component feasible?	Y p. 8		
2 Is the qualitative component feasible?	Y p. 6–7		
3 Is the mixed methods design feasible?	Y p. 5		
4 Have both qualitative and quantitative components been completed?	Y p.12–16		
5 Were some quantitative methods planned but not executed?		*N*	
6 Were some qualitative methods planned but not executed?		*N*	
7 Did the mixed methods design work in practice?	Y Findings Risk nursing diagnosis p.12–13, Table 2		
Assessment of the mixed methods design of studies in HSR			
1 Is the use of mixed methods research justified?	y p. 4 (Corry Model, development of a nursing diagnosis) y p.5 Design		
2 Is the design for mixing methods described? Priority Purpose Sequence Stage of integration	y p.5–6 (Figure 3), 9 (data integration)		
3 Is the design clearly communicated?	Y p. 5		
4 Is the design appropriate for addressing the research questions?	y p. 4–5		
5 Has rigor of the design been considered (proposal) or adhered to (report)?	y p. 9, p.11		
Assessment of the quantitative component of mixed methods studies in HSR			
1 Is the role of each method clear?	y p. 4 (research question), p. 5 (Figure 3), p. 9 (data integration)		
2 Is each method described in sufficient detail?	y p5−10		
3 Is each method appropriate for addressing the research question?	y p. 4–5		
4 Is the approach to sampling and analysis appropriate for its purpose?	y p. 6–7		
5 Is there expertise among applicants/authors?	Y p. 7 (data collection through APNs) 10 (rigor)		
6 Is there expertise on the team to undertake each method?	y p. 10 (rigor)		
7 Have issues of validity been addressed for each method?	Y p. 8 (interrater reliability) 10 (inference validity)		
8 Has the rigor of any method been compromised?		*N*	
9 Is each method sufficiently developed for its purpose?	y p. 7–9		
10 Is the (intended) analysis sufficiently sophisticated?	y p. 7–10		
Assessment of the qualitative component of mixed methods studies in HSR			
1 Is the role of each method clear?	y p. 5, 6, Figure 3		
2 Is each method described in sufficient detail?	Y p. 5–7		
3 Is each method appropriate for addressing the research question?	Y p. 5 (research question), p. 6–10 (Method), 11–15 (Findings)		
4 Is the approach to sampling and analysis appropriate for its purpose?	y p. 6–7 (Method)		
5 Is there expertise among the applicants/authors?	y p. 7 (APN), p. 10 (supervisor, senior researcher)		
6 Is there expertise on the team to undertake each method?	Y p. 7–10		
7 Have issues of validity been addressed for each method?	y p. 7–8 (collection of data), 10 (rigor)		
8 Has the rigor of any method been compromised?		*N*	
9 Is each method sufficiently developed for its purpose?	y p. 6–7 (Methods observations, interviews)		
10 Is the (intended) analysis sufficiently sophisticated?	y p. 6–7		
Table 5 Assessment of integration in mixed methods studies in HSR			
1 Is the type of integration stated?	y p. 9		
2 Is the type of integration appropriate to the design?	y p. 9		
3 Has enough time been allocated for integration?	y p. (1 year)		
4 Is the approach to integration detailed in terms of working together as a team?	y p. 9–10 (inference validity, research group)		
5 Does the dissemination strategy detail how the mixed methods will be reported in final reports and peer‐reviewed publications?	y current publication J Nursing Open, NANDA‐I		
6 Are the personnel who participate in the integration clearly identified?	y p. 10–11 (academic society, co‐author, supervisor…)		
7 Did appropriate members of the team participate in integration?	Y p. 10		
8 Is there evidence of communication within the team?	Y p. 10–11 (rigor)		
9 Has rigor been compromised by the process of integration?		*N*	
