# Supplementary material for: Developing a nursing diagnosis for the risk for malnutrition: a mixed‐method study
Source: Nurs Open. 2021 Jan 21;8(3):1463–78. doi: 10.1002/nop2.765 (PMC8046117; doi:10.1002/nop2.765)
Supplement: Supplementary file 1 — Supplementary Material [file NOP2-8-1463-s001.docx]

**SUPPORTING INFORMATION**

*Supp.1: Observation guide*

1. Where does the person in charge of the Nutrition and Service (TWS) team obtain information on the prescribed diet and any special requirements such as portion size (e.g. 1/2 portion or high energy) - observe the TWS's skills
2. When is a diet prescribed? And by whom?
3. Doctor's visit: Is nutrition an issue
4. To what extent are diet and patient nutritional status taken into account by the TWS? (e.g. whether a snack between meals is indicated) - TWS can be asked
5. Is nursing information on the deficit of self-sufficiency in eating and drinking (difficulty swallowing, oral hygiene) read?
6. Which services are offered? How vast is the selection? (Number of menu suggestions / variations? (Look at the menu, which is allowed for the patient.)
7. How and by whom is the wish to have food expressed?
8. How and by whom is food ordered?
9. Food distribution: What is the time interval between the acoustic signal and the food being served to the patient?
10. Is the distribution of food under deliberate control? (e.g. dipl. PP goes to patient suffering from dysphagia who needs help)
11. Who brings the food? To the observed patient?
12. Does the food on the tray match the menu card?
13. Is support offered for personal hygiene before eating (mouthwash, face wash, hand wash, use dental prosthesis, clean glasses)?
14. What is done for stimulating the appetite? (e.g. ambiance, clean table, ventilate room...)
15. Place of meals consumption
16. How are patients seated?
17. How is the food served (e.g. cover the tray, open the butter, reach the food and place it in the right position for service, etc.); how is it arranged (visually)?
18. How are the patients supported during meal?
19. Is the support adapted to the need of care (food intake time, time for food intake in case of swallowing disorder)?
20. In what way is food fed? Is the feeding careful and dignified?
21. How is food assistance completed? (e.g.: Oral care? Other position? Enquiry)
22. Where are snacks stored?
23. When are snacks distributed (e.g. yoghurt, liquid food) - in the evening, a check-up with the patients and in the refrigerator of the kitchen!
24. Who distributes snacks between meals?
25. How are snacks served?
26. By whom, when and where the consumption of the snack is documented?
27. In between, what "little things" does the patient eat on his own?
28. Who (role?) clears the food tray out of the room?
29. What does the cleaner do?
30. How is eating behaviour and eating (drinking) quantity monitored? (e.g. is the plate looked at, inquired?)
31. Does liquid food or fortified food remain untouched?
32. Who documents the amount of food?
33. Where is the amount of food documented?
34. Who documents the drinking quantity
35. Where is the drinking quantity documented (drinking protocol, monitoring sheet, meal cards, Medfolio nursing report)?
36. Who reports to whom regarding the diets
37. Who reports to whom in terms of food quantity
38. Who reports to whom regarding nutritional status (weight, how many patients have eaten...) (doctor’s ward round, interdisciplinary reports, shift hand over between registered nurses and others)
39. At the end of the early shift (approx. 14:30), ask the ward manager:

A) Whether something about nutrition (problems, self-sufficiency) is documented in the nursing plan or anamnesis of the observed patient.

B) Whether a snack is documented in the Medfolio under prescriptions or in the nursing report.

1. Other observations in connection with food?

*Supp. 2: Semi-structured interview schedule*

1. Please describe your experience with ordering meals?
2. How do you perceive the preparation for food?
3. What are your observations about the process when the food tray is removed?
4. In which way is the consumed quantity of food and drink monitored?
5. Questions about your state of nutrition
6. During your hospital stay, did you express any wish regarding food?
7. Did you rise a claim about food served (quantity of food, choice and / or diet)?
8. Do you have a food reluctance? (after the meeting check in Medfolio!) Do you have a food allergy?
9. How well do you feel nourished?
10. How satisfied are you with the food during your hospital stay?
11. Is there anything else regarding food or nutrition you would like to share with me?

Demographic data

1. Number of school and higher education years
2. Former profession?
3. Do you have to take care of relatives, pets or plants in your familiar environment?

Supporting Table 1 (Supp.T1) Theory of NANDA-I standards to develop a nursing diagnosis (Supplementary material)

| **nursing diagnosis according to NANDA-I consist of three parts** | **NANDA-I nursing diagnosis consist of seven axes** | **Eight levels of evidence of NANDA-I nursing diagnosis** |
| --- | --- | --- |
| (a) a label with a diagnosis title and a definition  (b) defining characteristics or risk factors  (c) associated conditions  (d) at-risk population  (Herdmann & Kamitsuru, 2018) | (1) focus of the diagnosis such as the main human response on health status  (2) subject (individual)  (3) judgement (impaired)  (4) location (oral)  (5) age (oldest-old)  (6) time (acute)  (7) status (risk)  (Herdmann & Kamitsuru, 2018, p. 99) | LOE 1.1: *Label only* (literature-based *label*, title)  LOE 1.2: *Label and definition* of a human response (consistent with the NANDA-I general definition of nursing diagnoses, and supported by literature)  LOE 1.3.: *Theoretical level* (references are cited, and facultatively expert opinion is used)  LOE 2.1: *Label, definition, defining characteristics and related factors, or risk factors, and references* (with additional links to nursing interventions and nursing outcomes from a standardized nursing terminology)  LOE 2. 2: *Concept analysis:* Criteria for LOE 2.1. are met (and a narrative review with a concluding *concept analysis* supports the value of label, definition and risk factors)  LOE 2.3: *Consensus using experts* (the beforementioned characteristics are met, and soliciting expert opinion e.g., consensus with Delphi or similar method with nurses as experts is performed)  LOE 3.1: *Clinically supported validation with literature synthesis:* LOE 2.2. are met (and the synthesis is an integrated review of the literature based on MeSH terms)  LOE 3.2. *Clinical study related to the diagnosis, but not generalizable:* LOE 2.2. are met (the narrative includes a description of studies related to the diagnosis, which includes risk factors. Studies may be qualitative in nature, or quantitative using non-random samples in which patients are subjects) |

*Supp.T2*

**Validated instrument for quantitative data collection**

*Q-DIO N, as adapted with the agreement of the instrument developer M. Müller-Staub* (Müller-Staub et al., 2008, p. 23table 1; Müller‐Staub, Needham, Odenbreit, Lavin, & Van Achterberg, 2007)

| Dimension | Scoring: 0, 1 or 2 maximal sum-score per dimension |
| --- | --- |
| **Nursing Diagnoses as Process**  01 Actual situation, leading to the hospitalization  02 Anxiety and worries related to hospitalization, expectations and desires about hospitalization  03 Social situation and living environment/circumstances  04 Coping in the actual situation/with the illness  05 Beliefs and attitudes about life (related to the hospitalization)  06 Information of the patient and relatives/significant others about the situation  07 Intimacy, being female/male  08 Hobbies, activities for leisure  09 Significant others (contact persons)  10 Activity of daily living: *to eat and drink, consider 1^st^ Nutrition risk Score* since admission  11 Relevant nursing priorities according to the assessment (focus on *to eat and drink*) | 22 |
| **Nursing Diagnoses as Product**  12 The etiology (E) is documented  13 The etiology (E) is correct, related/corresponding to the nursing diagnosis (P)  14 Signs and symptoms are formulated  15 Signs and symptoms (S) are correctly related to the nursing diagnosis (P)  16 The nursing goal relates/corresponds to the nursing diagnosis  17 The nursing goal is achievable through nursing interventions | 12 |
| **Nursing Interventions**  18 Concrete, clearly named nursing interventions are planned (what will be done, how, how often, who does it)  19 The nursing interventions affect the etiology of the nursing diagnosis  20 Nursing interventions carried out, are documented | 6 |
| **Nursing-Sensitive Patient Outcomes**  21 Acute, changing diagnoses are assessed daily or form shift to shift/enduring diagnoses are assessed every fourth day  22 The nursing diagnosis is reformulated  23 The nursing outcome is documented  24 The nursing outcome is observably/measurably documented  25 The nursing outcome shows  – improvement in patient’s symptoms  – improvement of patient’s knowledge state  – improvement of patient’s coping strategies  – improved self-care abilities  – improved functional status  26 There is a relationship between outcomes and nursing interventions  27 Nursing outcomes and nursing diagnoses are internally related (outcome is related to problem and etiology and symptoms (PES) | 14 |
| **Total maximal sum score** | 54 |

0 = not documented, 1 = one aspect is documented with medium quality, 2 = two or more aspects are documented and comprehensively recorded in good quality


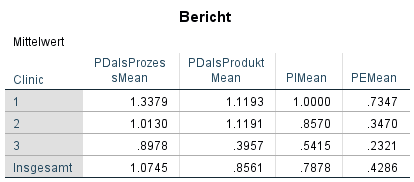


Supp. T3 Footnote: ND=Nursing diagnosis, 1= geriatric care ward, 2=perioperative care, 3=intern medicine

ND process ND product intervention outcome

Supp.T 3 Q-DIO Mean sum score per clinic and dimension

Total

Supp.T 4 Q-DIO mean, standard deviation, standard error, confidence interval, minimal and maximal scores


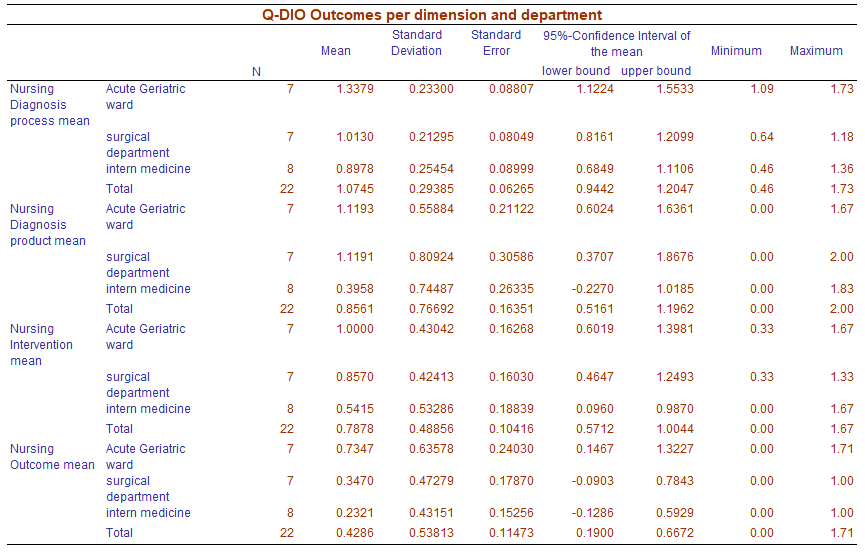


Supp.T4 Footnote: surgical department=perioperative ward

Supp. T5

*Summary of reviews with codes of risk factors (main outcomes)*

| **Author (Date) Journal / country** | **Title** | **Design, study duration** | **Subject of investigations** (aim “to investigate”) | **Setting** | **Number of participants, Sampling (y)** | **Elaborated risk factors** |
| --- | --- | --- | --- | --- | --- | --- |
| Namasivayam AM, Steele CM. (2015) J Nutr Gerontol Geriatr. / US, ES, FI, DE | Malnutrition and Dysphagia in long-term care: a systematic review | Systematic review,  1 month, November 2013, publication dates 1946-2013 | The impact of dysphagia on malnutrition | Long-term care, multicenter, 14 studies included | Age > 51y., in most studies > 70 y. | Impaired swallowing |
| Nieuwenhuizen et al. (2010). Clin Nutr,/ country: N/A | Older adults and patients in need of nutritional support: review of current treatment options and factors influencing nutritional intake. | Review  Publication dates until December 2008, (N/A for study duration) | Factors that influence nutritional intake in older adults | N/A | 123 publications, “elderly”, “older adults” | **Product related:** Ingredients - high protein, high fibre, low-digestible carbohydrates  Food attributes: High viscosity; large volume; monotonous diets; culturally inappropriate food; too large portion size  **Personal related:** low socio-economic status; physiological (most frequent: cancer, cardiac disorders); psychiatric factors (depression); mealtime environment  **Environmental:** Living alone; social isolation; inappropriate mealtime environment (disruptions, eating hours); care dependency (lack of help with eating) |
| Hasseler M. (2010) Pflege Z. / country: N/A | Malnutrition in the elderly in health care. Causes, instruments and consequences | Review, publication dates 2000-2009 | Causes and associated factors of malnutrition | Any health care institution | Publications from 2000-2009 | Age 80 and over and ageing-related factors such as gastro-intestinal dysfunction, impaired oral cavity status, neurocognitive disorder (dementia, delirium), loneliness, losses, or sensory dysfunction leading to appetite loss, food and fluid intake, and changed absorption of nutrients. |

Supp.T6

*Summary of exploratory studies with codes of risk factors (main outcomes) (Reduction II according to Mayring (abbreviations^[[1]](#footnote-1)^)*

| **Author (date), journal / country** | **Title** | **Design, duration** | **Setting** | **Number, sampling (mean age in years, y.)** | **Elaborated risk factors** |
| --- | --- | --- | --- | --- | --- |
| Galesi et al. (2013) Int J Older People Nurs. / BR | Association between indicators of dementia and nutritional status in institutionalized older people. | N/A | Single-center, LTC | 150 institutionalized older people, stratified probability, (77y.) | Dementia and female gender |
| Bonetti et al. (2017) J Clin Nurs. / IT | [Prevalence of malnutrition among older people in medical and surgical wards in hospital and quality of nutritional care: A multicenter, cross-sectional study.](https://www.ncbi.nlm.nih.gov/pubmed/28833723) | Observation, cross-sectional, study, 1-day | Multicenter (12 hospitals), medical and surgical hospital units | 1066 patients, convenience, “all patients in one day” (76.8y.) | Female gender; > 3 comorbidities, aged ≥ 85y, pressure ulcers, impaired autonomy, taking ≥ 3 medications |
| Chen et al. (2010) Nurs Res. / TW | Shared risk factors for distinct geriatric syndromes in older Taiwanese inpatients. | Observation, cross-sectional, 5 rounds of 24-unit visits (year or months of data collection not given) | Single-centered, medical center, 24 medical and surgical units | 455 patients, randomized (75.3y.) | Female gender  Care dependency (lower score of Barthel Index)  Neurocognitive disorder (MMSE),  Psychiatric factors (depression, GDS) |
| Chen et al. (2009) J Clin Nurs. / TW | Trajectory and determinants of nutritional health in older patients during and six-month post-hospitalization. | Observation, prospective cohort study, follow-up with 4 time points: within 48 hours after admission, before discharge and 3–6 months post-discharge, during 1.5 y (2004-2006) | Single-centered, tertiary hospital | 306 patients, clustered purposive (71.8y.) | Acute disease and or hospitalizations  Female gender, widowhood, age (65 years and above), low socio-economic status, polypharmacy and/or multimorbidity, impaired oral cavity status, neurocognitive disorder, care dependency, psychiatric factors, social isolation |
| Chen et al. (2007) J Clin Nurs. / TW | Revisiting the concept of malnutrition in older people. | Observational design, concept-evaluation, seeking correlation, 6 months data collection (March-August 2004) | Single-centered, cardiac and orthopedic services at one tertiary hospital | 114 patients, convenience (75.2y.) | Polypharmacy and/or multimorbidity, female gender, sensory decline, psychiatric factors (depressive symptoms); age (65 years above), impaired oral cavity status, neurocognitive disorder |
| Lindorff-Larsen et al. J et al. (2007) Clin Nutr / DK | Management and perception of hospital undernutrition - A positive change among Danish doctors and nurses | Pre-post-test design, 2 time points: 1997 and 2004 | Multi-center, hospitals, community care | 1145 doctors and nurses, randomized, (56% of participating doctors and nurses were >40y.) | Health-care workers attitude and culture – missing awareness (lack of knowledge; interest and defined responsibility; lack of documentation (of effect); Time consuming; difficulty to identify relevant patients |
| Mudge et al. (2011) Clinical Nutrition / AU | Helping understand nutritional gaps in the elderly (HUNGER): A prospective study of patient factors associated with inadequate nutritional intake in older medical inpatients | Prospective cohort study, multi-method-study, Nov 2007-March 2008; 4 months | Single-centered, 4 acute general medicine wards | 134 patients, convenience (80y.) | age (65 years above), appetite loss, care dependency (required help with set-up, supervision or actual feeding) and body composition (obesity) |
| Müller et al. (2017) Eur J Clin Nutr. / CH | Impaired nutritional status in geriatric trauma patients. | Observational, cohort study, March-June 2016, 4 months | Single-centered, geriatric trauma ward, tertiary hospital | 169 patients convenience, (78y. Male -81y. Female) | psychiatric factors (GDS), neurocognitive disorder, body composition (prevalent frailty), polypharmacy and/or multimorbidity |
| Patel MD, Martin FC. (2008) J Nutr Health Aging. / GB | Why don't elderly hospital inpatients eat adequately? | Exploratory, longitudinal study, May 1999-Feb 2000, 10 Months | Single-centered, inner-City teaching Hospital | 100 patients convenience (81.7y.) | Acute disease, impaired oral cavity status, Impaired swallowing,  Psychiatric factors (mood / anxiety / confusion), body composition (anorexia)  inappropriate mealtime environment (Catering limitations) |
| Peng et al. (2015) J Nurs Res. / TW | Cognition and social-physiological factors associated with malnutrition in hospitalized older adults in Taiwan. | Descriptive and cross-sectional, 2009 - 2011, 2 y. | Single center, general hospital | 401 patients, cohort (purposive) | neurocognitive disorder, female gender, Age (65 years and above), polypharmacy and/or multimorbidity, care dependency |
| Pirlich et al. (2005) Nutrition / DE | Social risk factors for hospital malnutrition | Observation (N/A for study duration) | Multicenter, 2 hospitals | 794 patient’s convenience (59.4y.) | Age (≥80 y), psychiatric factors (alcohol intake), social isolation, low socio-economic status; polypharmacy and/or multimorbidity (incl. chronic pain), care dependency (immobility or difficulty in preparing food), impaired oral cavity status, impaired swallowing |
| Rubenstein et al. (2001) J Gerontol A Bio Sci, Med Sci. / FR, ES, US | Screening for undernutrition in geriatric practice: developing the short-form Mini-Nutritional Assessment (MNA-SF) | Exploratory, correlational study, instrument development (N/A for study duration) | Multicenter, Multilevel: community dwelling | 155 patients, randomized (76.5y.) | Care dependency (mobility), psychiatric factors (stress or depression), acute disease, number of meals per day, body composition (Body Mass Index, weight loss), neurocognitive disorder (dementia), Polypharmacy and/or multimorbidity appetite loss. |
| Schrader et al. (2014); J Nutr Health Ageing / DE | Nutritional status according to Mini Nutritional Assessment is related to functional status in geriatric patients—independent of health status | Exploratory cross-sectional, August 2003-April 2004 (9 months) | Single-Centre, community hospital | 205 patients, convenience (82y.) | Psychiatric factors (depression), care dependency (lower physical function) |
| Söderhamn et al. (2011) J Clin Nurs. / NO | Perceived health and risk of undernutrition: a comparison of different nutritional screening results in older patients. | Cross-sectional design, Observation  November 2008–April 2009 (6 months) | Multi-centered, of 2 hospitals, 3 medical wards | 158 patients, convenience (78y.) | Psychiatric factors (helplessness, not satisfied with life) |
| Söderström, et al. (2013) J Clin Nutr / SE | Mealtime habits and meal provision are associated with malnutrition among elderly patients admitted to hospital | Cross-sectional study, March 2008-May 2009, (15 months) | Single-centered, county hospital, medical, surgical and orthopedic wards | 1771 patients, convenience (78.9y.) | Inappropriate mealtime environment (Organizational or structural factors: overnight fast ≥ 11h, fewer than four eating episodes a day), care dependency (not cooking independently), Age > 80 years, polypharmacy or multimorbidity (infectious disease or COPD or rheumatoid arthritis)  Neurocognitive disorder, |
| Volkert et al. (2010) J Nutr Health Aging. / DE | Undiagnosed malnutrition and nutrition‐related problems in geriatric patients. | Cross-sectional study  August 2003-April 2004 (9 months) | Single-center, community hospital, geriatric ward | 205 patients, randomized (83y.) | Hospitalization combined with age (65 years and above); body composition (weight-change) and appetite loss |
| Jacobsen et al. (2016) BMJ Open. / NO | Prevalence of factors associated with malnutrition among acute geriatric patients in Norway: a cross-sectional study. | Cross-sectional  2014-2015, 1y, | Multi-centered, 2 hospitals, 2 acute geriatric wards, cardiology and stroke | 120 patients, convenience (82.5y.) | Care dependency (low physical performance), body composition (sarcopenia), polypharmacy and/or multimorbidity (cancer, pulmonary disease) |

*Supp.T7 JBI questions for critical appraisal of systematic reviews*

Q1 Is the review question clearly and explicitly stated?

Q2 Were the inclusion criteria appropriate for the review question?

Q3 Was the search strategy appropriate?

Q4 Were the sources and resources used to search for studies adequate?

Q5 Were the criteria for appraising studies appropriate?

Q6 Was critical appraisal conducted by two or more reviewers independently?

Q7 Were there methods to minimize errors in data extraction?

Q8 Were the methods used to combine studies appropriate?

Q9 Was the likelihood of publication bias assessed?

Q10 Were recommendations for policy and/or practice supported by the reported data?

Q11 Were the specific directives for new research appropriate? (Aromataris & Munn, 2017)

| *Supp. T8: Critical appraisal of (systematic) literature reviews 0=no, 1=yes, 2= unclear, 3=not applicable (JBI, 2017)* | | | | |  |  |  |  |  |  |  |  |
| --- | --- | --- | --- | --- | --- | --- | --- | --- | --- | --- | --- | --- |
| Author Year | Q1 | Q2 | Q 3 | Q4 | Q5 | Q6 | Q7 | Q8 | Q9 | Q10 | Q11 | No of criteria fulfilled |
| Namasivayam, Ashwini M., & Steele, Catriona M. (2015) | 0 | 0 | 2 | 1 | 2 | 2 | 2 | 1 | 2 | 1 | 1 | 4 |
| Nieuwenhuizen, W. F., Weenen, H., Rigby, P., & Hetherington, M. M. (2010) | 0 | 1 | 1 | 1 | 1 | 2 | 1 | 1 | 0 | 1 | 1 | 8 |
| Hasseler M. (2010) | 1 | 1 | 2 | 1 | 2 | 0 | 2 | 2 | 2 | 1 | 1 | 5 |

(Aromataris & Munn, 2017)

*Supp.T9: JBI questions for Critical appraisal of non-randomized studies*

Q1.  Is it clear in the study what is the cause’ and what is the ‘effect’ (i.e. there is no confusion about which variable comes first)?

Q 2. Were the participants included in any comparisons similar?

Q3. Were the participants included in any comparisons receiving similar treatment/care, other than the exposure or intervention of interest?

Q4. Was there a control group?

Q5 5. Were there multiple measurements of the outcome both pre and post the intervention/exposure?

Q6. Was follow up complete and if not, were differences between groups in terms of their follow up adequately described and analysed?

Q7. Were the outcomes of participants included in any comparisons measured in the same way?

Q8. Were outcomes measured in a reliable way?

Q9. Was appropriate statistical analysis used?

Supp.T10

*Critical appraisal of non-randomized trials and exploratory designs 0=no, 1=yes, 2= unclear, 3=not applicable (JBI, 2017)*

| Author / (year of publication) | Q1 | Q2 | Q3 | Q4 | Q5 | Q6 | Q7 | Q8 | Q9 | No of criteria fulfilled |
| --- | --- | --- | --- | --- | --- | --- | --- | --- | --- | --- |
| Galesi LF, Leandro-Merhi VA, de Oliveira MR. (2013) | 1 | 0 | 0 | 0 | 0 | 1 | 1 | 1 | 1 | 5 |
| Bonetti L, Terzoni S, Lusignani M, Negri M, Froldi M, Destrebecq A. (2017) | 1 | 3 | 0 | 0 | 0 | 3 | 0 | 1 | 1 | 3 |
| Chen CC, Dai YT, Yen CJ, Huang GH, Wang C. (2010) | 1 | 3 | 0 | 0 | 0 | 3 | 0 | 1 | 1 | 3 |
| Chen CC, Tang ST, Wang C, Huang GH. (2009) | 1 | 3 | 3 | 0 | 3 | 1 | 1 | 1 | 1 | 5 |
| Chen CC-H, Bai YI, Huang GH & Tang ST (2007) | 0 | 3 | 3 | 0 | 3 | 1 | 3 | 1 | 1 | 3 |
| Lindorff-Larsen K, Rasmussen HH, Kondrup J et al. . Clin Nutr (2007) | 1 | 1 | 3 | 1 | 1 | 1 | 1 | 1 | 1 | 8 |
| Mudge A.M., Ross L.J., Young M.A., Isenring E.A. Banks M.D. (2011) | 1 | 3 | 3 | 0 | 0 | 1 | 0 | 1 | 1 | 4 |
| Müller FS, Meyer OW, Chocano-Bedoya P, Schietzel S, Gagesch M, Freystaetter G, Neuhaus V, Simmen HP, Langhans W, Bischoff-Ferrari HA. (2017) | 1 | 3 | 3 | 0 | 0 | 1 | 1 | 1 | 1 | 5 |
| Patel MD, Martin FC. J Nutr Health Aging. (2008) // Pilot-Study: M Patel, FC Martin. Why don’t elderly hospital inpatients eat adequately? (1999) | 1 | 3 | 0 | 1 | 2 | 2 | 0 | 1 | 1 | 4 |
| Peng LN, Cheng Y, Chen LK, Tung HH, Chu KH, Liang SY.J Nurs Res. (2015) | 1 | 3 | 3 | 0 | 0 | 1 | 1 | 1 | 1 | 5 |
| Pirlich M, Schutz T, Kemps M, Luhman N, Minko N, Lubke HJ, Rossnagel K, Willich SN & Lochs H (2005) | 1 | 3 | 3 | 0 | 0 | 1 | 0 | 1 | 1 | 4 |
| Rubenstein, L.Z., Harker, J.O., Salva, A., Guigoz Y., Vellas, B., (2001) | 1 | 1 | 1 | 1 | 1 | 1 | 1 | 1 | 1 | 9 |
| Schrader E, Baumgartel C, Gueldenzoph H, et al. J Nutr Health Ageing (2014) | 1 | 3 | 3 | 0 | 0 | 1 | 1 | 1 | 1 | 5 |
| Söderhamn U, Flateland S, Jessen L, Söderhamn O. (2011) | 1 | 3 | 0 | 0 | 0 | 2 | 1 | 1 | 1 | 4 |
| Söderström, L, Adolfsson E.T., Rosenblad A., Frid H., Saletti A., Bergkvist L. (2013) | 1 | 3 | 3 | 0 | 1 | 1 | 0 | 1 | 1 | 5 |
| Volkert D, Saeglitz C, Gueldenzoph H, Sieber CC, Stehle P. (2010) | 1 | 3 | 3 | 0 | 1 | 1 | 3 | 1 | 1 | 5 |
| Jacobsen EL, Brovold T, Bergland A, Bye A. (2016) | 1 | 1 | 1 | 1 | 1 | 1 | 1 | 1 | 1 | 9 |

**References**

Aromataris, E., & Munn, Z. . (2017). Joanna Briggs Institute Reviewer's Manual. 4. Retrieved from https://reviewersmanual.joannabriggs.org/

Herdmann, T. H., & Kamitsuru, S. (2018). *Nursing Diagnosis. Defintions and Classification 2018-2020* (11 ed.). New York: Thieme.

JBI, The Joanna Briggs Institute. (2017). *Joanna Briggs Institute Reviewer's Manual.* In E. Aromataris & Z. Munn (Eds.). Retrieved from https://reviewersmanual.joannabriggs.org/

Müller-Staub, M., Lunney, M., Lavin, M. A., Needham, I., Odenbreit, M., & van Achterberg, T. (2008). Testing the Q-DIO as an instrument to measure the documented quality of nursing diagnoses, interventions, and outcomes. *Int J Nurs Terminol Classif, 19*(1), 20-27. doi:10.1111/j.1744-618X.2007.00075.x

Müller‐Staub, Maria, Needham, Ian, Odenbreit, Matthias, Lavin, Mary Ann, & Van Achterberg, Theo. (2007). *Evaluation of the implementation of nursing diagnostics.*

*A study on the use of nursing diagnoses, interventions and outcomes in nursing documentation.* (18), Radboud University Nijmegen, The Netherlands. Retrieved from https://onlinelibrary.wiley.com/doi/abs/10.1111/j.1744-618X.2007.00043.x

http://hdl.handle.net/2066/40179 (1)

1. NRS, nutrition risk score according to Kondrup; sex: m, male, f, female, LOS, length of stay, DM2, diabetes mellitus type 2, doc., documentation [↑](#footnote-ref-1)
